# Melatonin Attenuates Dasatinib-Aggravated Hypoxic Pulmonary Hypertension via Inhibiting Pulmonary Vascular Remodeling

**DOI:** 10.3389/fcvm.2022.790921

**Published:** 2022-03-24

**Authors:** Rui Wang, Jinjin Pan, Jinzhen Han, Miaomiao Gong, Liang Liu, Yunlong Zhang, Ying Liu, Dingyou Wang, Qing Tang, Na Wu, Lin Wang, Jinsong Yan, Hua Li, Yuhui Yuan

**Affiliations:** ^1^The Second Affiliated Hospital, Institute of Cancer Stem Cell, Dalian Medical University, Dalian, China; ^2^Liaoning Key Laboratory of Hematopoietic Stem Cell Transplantation and Translational Medicine, Liaoning Medical Center for Hematopoietic Stem Cell Transplantation, Dalian Key Laboratory of Hematology, Second Hospital of Dalian Medical University, Dalian, China; ^3^College of Pharmacy, Dalian Medical University, Dalian, China; ^4^The First Affiliated Hospital, Dalian Medical University, Dalian, China

**Keywords:** melatonin, dasatinib, pulmonary hypertension, vascular remodeling, hypoxia, oxidative stress

## Abstract

Dasatinib treatment is approved as first-line therapy for chronic myeloid leukemia. However, pulmonary hypertension (PH) is a highly morbid and often fatal side-effect of dasatinib, characterized by progressive pulmonary vascular remodeling. Melatonin exerts strong antioxidant capacity against the progression of cardiovascular system diseases. The present work aimed to investigate the effect of melatonin on dasatinib-aggravated hypoxic PH and explore its possible mechanisms. Dasatinib-aggravated rat experimental model of hypoxic PH was established by utilizing dasatinib under hypoxia. The results indicated that melatonin could attenuate dasatinib-aggravated pulmonary pressure and vascular remodeling in rats under hypoxia. Additionally, melatonin attenuated the activity of XO, the content of MDA, the expression of NOX4, and elevated the activity of CAT, GPx, and SOD, the expression of SOD2, which were caused by dasatinib under hypoxia. *In vitro*, dasatinib led to decreased LDH activity and production of NO in human pulmonary microvascular endothelial cells (HPMECs), moreover increased generation of ROS, and expression of NOX4 both in HPMECs and primary rat pulmonary arterial smooth muscle cells (PASMCs) under hypoxia. Dasatinib up-regulated the expression of cleaved caspase-3 and the ratio of apoptotic cells in HPMECs, and also elevated the percentage of S phase and the expression of Cyclin D1 in primary PASMCs under hypoxia. Melatonin ameliorated dasatinib-aggravated oxidative damage and apoptosis in HPMECs, meanwhile reduced oxidative stress level, proliferation, and repressed the stability of HIF1-α protein in PASMCs under hypoxia. In conclusion, melatonin significantly attenuates dasatinib-aggravated hypoxic PH by inhibiting pulmonary vascular remodeling in rats. The possible mechanisms involved protecting endothelial cells and inhibiting abnormal proliferation of smooth muscle cells. Our findings may suggest that melatonin has potential clinical value as a therapeutic approach to alleviate dasatinib-aggravated hypoxic PH.

## Introduction

Dasatinib is one kind of second-generation multiple tyrosine kinase inhibitors primarily used to treat patients with chronic myeloid leukemia (CML) and Philadelphia chromosome-positive acute lymphoid leukemia (Ph^+^ ALL). For most CML and Ph^+^ ALL patients, dasatinib has made this potentially devastating disease into a manageable chronic condition. However, pulmonary hypertension (PH) is a recognized side-effect of dasatinib that occurs at any time during the treatment course, ranging from months to over one year (1–3). Although some functional and clinical improvements of patients were observed after cessation of dasatinib, the majority of patients failed to recover completely, and some died of PH-associated cardiac failure or even suffered from sudden death, suggesting that pulmonary vascular remodeling caused by dasatinib is the key clinical issue of dasatinib-associated PH (4, 5).

Pulmonary hypertension is a lethal disease featured by progressive pulmonary vascular remodeling that contributed to the obliteration of distal pulmonary arteries and elevated pulmonary artery pressure, leading to the right ventricle (RV) hypertrophy, eventually to heart failure (1, 6, 7). Pulmonary artery endothelial dysfunction and aberrant smooth muscle cells (SMCs) proliferation are involved in the pathological development of vascular remodeling. Recent studies have demonstrated that the impact of dasatinib on pulmonary endothelial cells (ECs) contributed substantially to PH, mainly through inducing mitochondrial reactive oxygen species (ROS) production, endoplasmic reticulum stress, and ECs apoptosis (8). ROS is able to stabilize hypoxia-inducible factor 1-α (HIF1-α) protein expression (9), HIF-1α functions as an intrinsic pathogenic determinant in many types of PH, including monocrotaline-induction and genetic forms (10, 11). Whereas, there is limited evidence about HIF-1α in dasatinib-associated PH and a lack of drugs targeting for pulmonary vascular remodeling of PH in dasatinib-treated patients, and no cure for this devastating disease at present (12, 13). Thereby, it is significant to develop effective drugs for attenuating vascular remodeling against dasatinib-associated PH.

Oxidative stress is regarded to play a crucial role in vascular remodeling of PH (14–17). In this context, natural antioxidants have been used as treatments for PH. Melatonin (N-acetyl-5-methoxytryptamine) is a small lipophilic molecule and ubiquitous physiological mediator mainly synthesized in pineal gland (18). As a neurohormone, melatonin is characterized with pleiotropic activities, such as treating insomnia (19), inhibiting tumor growth (20, 21), and being utilized as an adjuvant of cancer therapies (22, 23). Numerous investigations have indicated that melatonin also has many benefits on the cardiovascular system, including regulation of atherosclerosis, depression of hypertension, and prevention of hypertensive heart disease (24–26). Moreover, melatonin alleviated hemodynamics and pulmonary vascular remodeling in hypoxic PH rats (18), decreased pulmonary vascular remodeling and oxygen sensitivity in pulmonary hypertensive newborn lambs (16), and alleviated vascular disorders in monocrotaline-induced PH rats (27). Nevertheless, the efficacy of melatonin on dasatinib-associated PH has not been investigated so far.

In the present study, we explored the effect and the possible underlying mechanism of melatonin on dasatinib-aggravated PH. Melatonin treatment blunted pulmonary vascular remodeling and significantly attenuated hemodynamics in a rat model of dasatinib-aggravated PH under normobaric hypoxic condition. *In vitro* findings suggest that melatonin may reduce pulmonary vascular remodeling by protecting ECs and inhibiting proliferation of SMCs.

## Materials and Methods

### Animal Experiments

Adult male Sprague–Dawley (SD) rats (6–8 weeks) weighing from 120 to 150 g were obtained from the Animal Center of Dalian Medical University (Dalian, China). All procedures were carried out following the Institutional Animal Care and Use Committee guidelines, and approved by the Institutional Ethics Committee.

Dasatinib-aggravated experimental model of hypoxic PH was established as previously described (8). Forty-eight adult male SD rats were randomly divided into six groups (*n* = 8): (1) vehicle under normoxia (Nor + Vehicle) group, (2) dasatinib under normoxia (Nor + Das) group, (3) melatonin under normoxia (Nor + Mel) group, (4) vehicle under hypoxia (Hyp + Vehicle) group, (5) dasatinib under hypoxia (Hyp + Das) group, and (6) dasatinib with melatonin administration under hypoxia (Hyp + Das + Mel) group.

Normoxia groups rats were maintained in room air, hypoxia groups rats were kept in chambers for normobaric hypoxia in the same room and had free access to water and regular chow. The oxygen fraction inside the chamber was maintained at 10% flushed with N_2_ and room air. The hypoxia groups rats underwent normobaric hypoxic condition (10% O_2_, 12 h/day) at day14 for 3 weeks. Nor + Das group rats were treated with daily i.p. injection of dasatinib (Selleck, United States) at a dose of 10 mg/kg/day for 4 weeks, Hyp + Das and Hyp + Das + Mel group rats were treated with daily i.p. injection of dasatinib for 1 week and received hypoxia induction for the next 3 weeks, Nor + Mel and Hyp + Das + Mel group rats were administrated with daily i.p. injection of melatonin (Sigma, United States) at a dose of 15 mg/kg/day for the entire 5 weeks.

### Echocardiography

Cardiopulmonary parameters, including cardiac output and velocity-time integral (VTI) were measured using non-invasive digital ultrasound micro-imaging system (Vevo 770 system VisualSonics, Canada) as previously described (28).

### Hemodynamic Experiments

Measurement of right ventricular systolic pressure (RVSP) was performed as described previously (18, 29). Blood samples were obtained from the heart into EDTA tubes (2 mL K^+^/EDTA), centrifuged at 3,000 rpm for 15 min, and plasma was kept at −80°C for subsequent experiments. The RV was separated from the left ventricle plus septum (LV + S), and the RV/(LV + S) ratio was calculated as the Fulton index. The Fulton index [RV/(LV + S)] and RV/body weight (BW) were calculated as RV hypertrophy. The lung tissues were dissected into 4-μm-thick slices and placed in 4% paraformaldehyde solution for 72 h. The left lungs were stored at −80°C for subsequent experiments.

### Morphological Investigation

The right lungs were fixed in 4% paraformaldehyde buffer at 4°C for 72 h, the midsagittal slices of right lungs were processed for paraffin embedding and sliced into 4-μm-thick sections. The sections were next subjected to hematoxylin and eosin (HE) staining according to the established techniques (18, 30). The percent medial wall thickness (WT%) = ([2 × medial wall thickness/external diameter] × 100), and percent medial wall area (WA%) = ([medial wall area/total vessel area] × 100) were calculated to access pulmonary vascular structure remodeling.

### Pulmonary Immunohistochemistry

Sections were dewaxed, rehydrated, retrieved the antigens, and treated with 1% H_2_O_2_ for 15 min at room temperature to block endogenous peroxidase. After incubated with 5% bovine serum albumin for 30 min, sections were treated with the primary antibodies against α-SMA (1:1,000, ab7817, Abcam, United States), PCNA (1:5,000, 2586, Cell Signaling Technology, United States) at 4°C overnight. Then, a biotinylated anti-mouse IgG antibody and an avidin-biotinylated peroxidase complex were applied with 3, 3-diaminobenzidine as a peroxidase substrate. Immunoreactivity was visualized by diaminobenzidine. A light hematoxylin counterstain was applied.

The integrated optical density (OD) of α-SMA was analyzed in the wall of the pulmonary arterioles, and the degree of pulmonary arteries muscularization was also determined by α-SMA staining. As previously described (30), 30–40 arteries were categorized as non-muscularized (α-SMA staining <25% of vessel circumference), partial muscularized (α-SMA staining 25–74% of circumference), or full muscularized (α-SMA staining >75% of circumference) vessels in each rat. The percentages of full muscularized vessels were calculated by dividing the number of vessels in the muscularized category by the total number counted in the same experimental group. Additionally, the numbers of PCNA-positive cells and all the cells in the pulmonary arterial media walls were counted. The percentage of PCNA-positive cell number was calculated as positive cells/all cells.

### Measurement of Plasma Melatonin Level

The quantitative determination of plasma melatonin concentrations was performed by enzyme-linked immunosorbent assay (E-EL-R0031c, Elabscience, China), according to the manufacturer’s guidelines. The lower limit of detection in this assay is 15.63 pg/mL, the intra-assay coefficient of variation (CV) is less than 5.24%, and the inter-assay CV is less than 5.30%.

### Rat Primary PASMCs Isolation and Cell Culture

Primary PASMCs were isolated from pulmonary arteries of adult male SD rats (6 weeks old) as previously described (18, 29). Cells from passages 3–6 were used for the *in vitro* studies. Primary PASMCs were cultured in High-glucose DMEM (Gibco, United States) containing 10% (v/v) fetal bovine serum (FBS), and antibiotics (penicillin 100 units/mL, and streptomycin 100 μg/mL).

Human pulmonary microvascular endothelial cells (HPMECs) were obtained from ScienCell. HPMECs were cultured in endothelial cell medium (ECM; ScienCell, United States) containing 10% FBS, endothelial cell growth supplement (ECGS; ScienCell, United States), and antibiotics.

### Cell Treatment

HPMECs or PASMCs were divided into six groups: (1) vehicle under normoxia (Nor + Vehicle) group, (2) dasatinib under normoxia (Nor + Das) group, (3) melatonin under normoxia (Nor + Mel) group, (4) vehicle under hypoxia (Hyp + Vehicle) group, (5) dasatinib under hypoxia (Hyp + Das) group, and (6) dasatinib and 10 μM melatonin under hypoxia (Hyp + Das + Mel) group. PASMCs at a density of 4 × 10^4^/mL, and HPMECs at a density of 8 × 10^4^/mL were seeded on 96-well plates or culture flask, and treated at the time of 70–75% confluence. Melatonin in FBS-free medium was added 1 h before exposure to 10 nM dasatinib or vehicle under normoxia (21% O_2_) or hypoxia (1% O_2_) condition for 24 h (18).

### Cell Viability Assay

Pulmonary arterial smooth muscle cells at a density of 4 × 10^4^/mL, HPMECs at a density of 8 × 10^4^/mL were seeded in 96-well plates, and cell viability was assessed by Cell Counting Kit-8 (CCK-8, Bimake, United States) following the manufacturer’s protocol. CCK-8 working solution (10 μL) was added to each well of the plate, and incubated the cells for 2 h in the incubator, then measured the absorbance at 450 nm by a microplate reader.

### Intracellular ROS Measurement

Intracellular ROS production was detected using DCFH-DA ROS probes (S0033S, Beyotime, China) (29). Briefly, cells were incubated with the probe (10 μM) for 30 min at 37°C in the dark, then rinsed three times to remove the excess probe and maintained in FBS-free DMEM medium. The fluorescence intensity of DCF was measured by CytoFLEX (Beckman Coulter, United States) in FL-1 channel (FITC).

### Apoptosis Assay

Annexin V Apoptosis Detection Kit (556420, BD Biosciences, CA, United States) containing Annexin V-FITC and propidium iodide (PI) was employed to measure the cell apoptosis rate. Measurements of the fluorescence were performed by CytoFLEX (Beckman Coulter, United States).

### Cell Cycle Assay

The synchronized PASMCs suspension was fixed with cold 75% ethanol at -20°C for 12 h, then stained with PI staining buffer at 37°C for 30 min, and detected by Accuri C6 plus Flow Cytometer (BD Biosciences, San Jose, CA, United States) in FL-2 channel (PE). As described previously (30), further analyses of cell cycle were processed by Flow Jo software.

### Xanthine Oxidase, Catalase, and Glutathione Peroxidase Activity Assay

Xanthine oxidase (XO) activity was determined by XO assay kit (BC1095, Solarbio, China), the intra-assay CV is less than 3%, and the inter-assay CV is less than 5%. According to the manufacturer’s instructions, the product of the reaction was quantified at an absorbance of 290 nm.

Catalase (CAT) activity was determined by catalase assay kit (S0051, Beyotime, China), the lower limit of detection in this assay is 1 U/mL, the intra-assay CV is less than 3%, and the inter-assay CV is less than 10%. According to the manufacturer’s instructions, the product of the reaction was quantified at an absorbance of 240 nm.

Glutathione peroxidase (GPx) activity was determined by total GPx assay kit with NADPH (S0058, Beyotime, China), the lower limit of detection in this assay is 0.5 mU/mL, the intra-assay CV is less than 3%, and the inter-assay CV is less than 10%. According to the manufacturer’s instructions, the product of the reaction was quantified at an absorbance of 340 nm.

### Superoxide Dismutase, Lactate Dehydrogenase Activity, and Malondialdehyde Content Assay

Superoxide dismutase (SOD) activity was determined by SOD activity assay kit (S0101S, Beyotime, China), the lower limit of detection in this assay is 0.5 U/mL, the intra-assay CV is less than 3%, and the inter-assay CV is less than 15%. According to the manufacturer’s instructions, the product of the reaction was quantified at an absorbance of 450 nm.

Lactate dehydrogenase (LDH) activity was determined LDH activity assay kit (C0016, Beyotime, China), the lower limit of detection in this assay is 0.65 mU/mL, the intra-assay CV is less than 3%, and the inter-assay CV is less than 10%. According to the manufacturer’s recommendations, the product of the reaction was quantified at an absorbance of 490 nm.

Malondialdehyde (MDA) content was determined by Micro-MDA assay kit (KGT003-1, Keygen, China), the lower limit of detection in this assay is 0.5 nmol/mL, the intra-assay CV is less than 2.3%, and the inter-assay CV is less than 5.4%. According to the manufacturer’s instructions, the product of the reaction was quantified at an absorbance of 532 nm.

### NO Generation Measurement

NO generation was measured using a fluorescence method. In this assay, 3-amino, 4-aminomethyl-2′, 7′-difluorescein, diacetate (DAF-FM-DA, S0019, Beyotime, China) was used as a fluorescent indicator of intracellular NO. Briefly, HPMECs were incubated with 5 mM DAF-FM-DA at 37°C for 20 min. Cells were then rinsed three times to remove the excess probe and maintained in phosphate-buffered saline (PBS) throughout the experiments. Fluorescence was recorded at excitation 495 nm/emission 515 nm on a fluorescence multi-well plate reader (Spark, Tecan Trading AG, Switzerland). Data were expressed as the fluorescence signal of the sample relative to the control.

### MitoSOX Red Staining

Pulmonary arterial smooth muscle cells were incubated with 5.0 μM MitoSOX Red Mitochondrial Superoxide Indicator (Yeasen, China) for 15 min to detect mitochondrial superoxide production using a fluorescent microscope (Olympus, Japan).

### Western Blotting Analysis

Protein expression in total lung tissues, HPMECs, and PASMCs were determined by Western blotting. After transferation, the PVDF membranes (Millipore, United States) were blocked with 5% skim milk to prevent non-specific binding, then incubated with specific primary antibodies against HIF1-α (1:500, ab216842, Abcam, United States), NOX4 (1:1,000, 14347-1-AP, Proteintech, China), SOD2 (1:1,000, 24127-1-AP, Proteintech, China), SOD1(1:1,000, 10269-1-AP, Proteintech, China), Cleaved Caspase-3 (1:1,000, 9664, Cell Signaling Technology, United States), Bcl-xL (1:1,000, AB126, Beyotime, China), β-actin (1:2,500, 60008-1-Ig, Proteintech, China). After washing with TBST buffer, the membranes were incubated with appropriate secondary antibody conjugated with horseradish peroxidase, and signals were detected using by enhanced chemiluminescent kit (Amersham Biosciences, United Kingdom). The relative OD of Western blotting was calculated with the Image lab software (Bio-Rad Laboratories, Hercules, CA, United States).

### Statistical Analyses

The mean values ± SEM were calculated and plotted using GraphPad Prism 7 software (GraphPad Software, San Diego, CA, United States). Differences between multiple groups with one variable were determined using by one-way ANOVA followed by Bonferroni’s *post hoc* test. To compare multiple groups with more than one variable were determined using two-way ANOVA followed by Bonferroni’s *post hoc* test was used. Significant difference was accepted at *P* < 0.05.

## Results

### Melatonin Attenuated Dasatinib-Aggravated Hypoxic Pulmonary Hypertension in Rats

**FIGURE 1:**
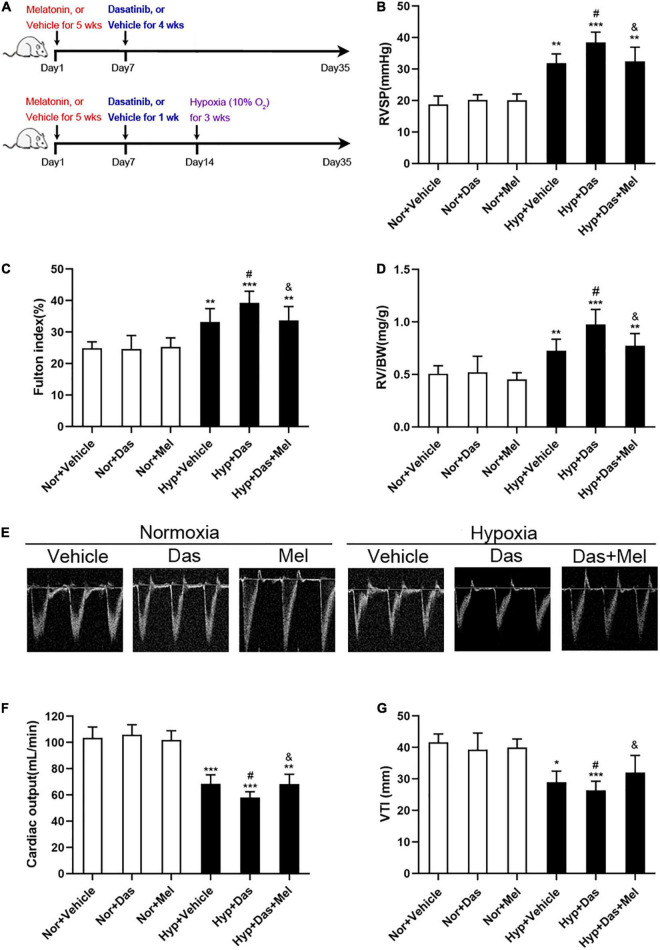
Melatonin attenuates pulmonary hypertension induced by dasatinib under chronic hypoxia. Schematic of rat model used to study the *in vivo* effects of melatonin on pulmonary hypertension induced by dasatinib under hypoxia **(A)**. Values of right ventricular systolic pressure (RVSP) **(B)**, Fulton index **(C)**, RV/BW **(D)**. Echocardiography of right ventricle **(E)**. Values of cardiac output **(F)** and VTI **(G)** in melatonin or vehicle pretreated rats which were exposed to dasatinib or vehicle under hypoxia or normoxia. Values are means ± SEM, ***P < 0.001, **P < 0.01, *P < 0.05 compared with Nor + Vehicle group. *^#^P* < 0.05 compared with Hyp + Vehicle group. *^&^P* < 0.05 compared with Hyp + Das group (*n* = 6).

To investigate the *in vivo* effects of melatonin on dasatinib-associated PH. Firstly, we established the dasatinib-aggravated experimental model of hypoxic PH (8). SD rats were treated with daily i.p. injection of dasatinib (10 mg/kg) or vehicle at day 7 for 1 week or 4 weeks, then administrated for PH induction: under chronic normobaric hypoxic condition (10% O_2_, 12 h/day) treatment (Hypoxia) for 3 weeks (as previously described). Before the establishment of dasatinib-aggravated PH model, rats were received daily i.p. injection of melatonin (15 mg/kg) for the entire 5 weeks (Figure 1A).

We found an increase in hemodynamics (right ventricle systolic pressure, RVSP) in Hyp + Das group rats (Figure 1B). In accordance with the RVSP, severer right ventricular hypertrophy (Fulton index, and RV/BW) (Figures 1C,D), lower cardiac output (Figure 1F) and VTI (Figure 1G) in Hyp + Das group, compared to Hyp + Das group rats. While the RVSP, Fulton index, RV/BW, cardiac output, and VTI in Hyp + Das + Mel group was reversed in the Hyp + Das + Mel group (Figures 1B–G).

### Melatonin Reduced Dasatinib-Aggravated Pulmonary Vascular Remodeling Under Hypoxia in Rats

**FIGURE 2:**
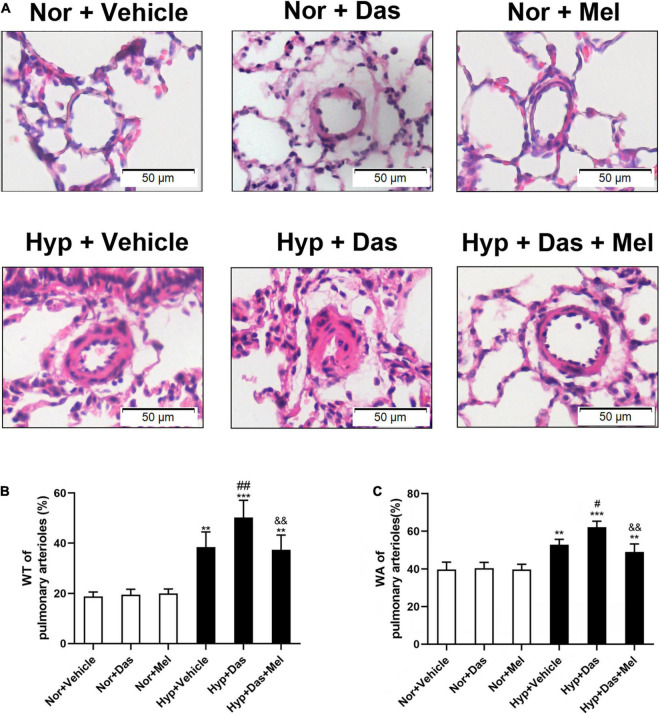
Melatonin attenuates vascular remodeling induced by dasatinib under hypoxia. Hematoxylin and eosin (HE) staining of pulmonary arterioles (original magnification ×20, scale bars: 50 μm.) **(A)**. Percentage of medial wall thickness (WT%) of pulmonary arterioles **(B)**. Percentage of medial wall area (WA%) of pulmonary arterioles **(C)**. Values are means ± SEM, ***P < 0.001, **P < 0.01 compared with Nor + Vehicle group. *^##^P* < 0.01, *^#^P* < 0.05 compared with Hyp + Vehicle group. *^&&^P* < 0.01 compared with Hyp + Das group (*n* = 6).

We next investigated the effects of melatonin on dasatinib-aggravated pulmonary artery remodeling in rats. The histological analyses showed that dasatinib produced a promotable effect on wall thickness (WT%) and wall area (WA%) of small pulmonary arteries in PH rats, while the WT% and WA% of Hyp + Das + Mel group rats were much lower than that of Hyp + Das group rats (Figures 2A–C).

**FIGURE 3:**
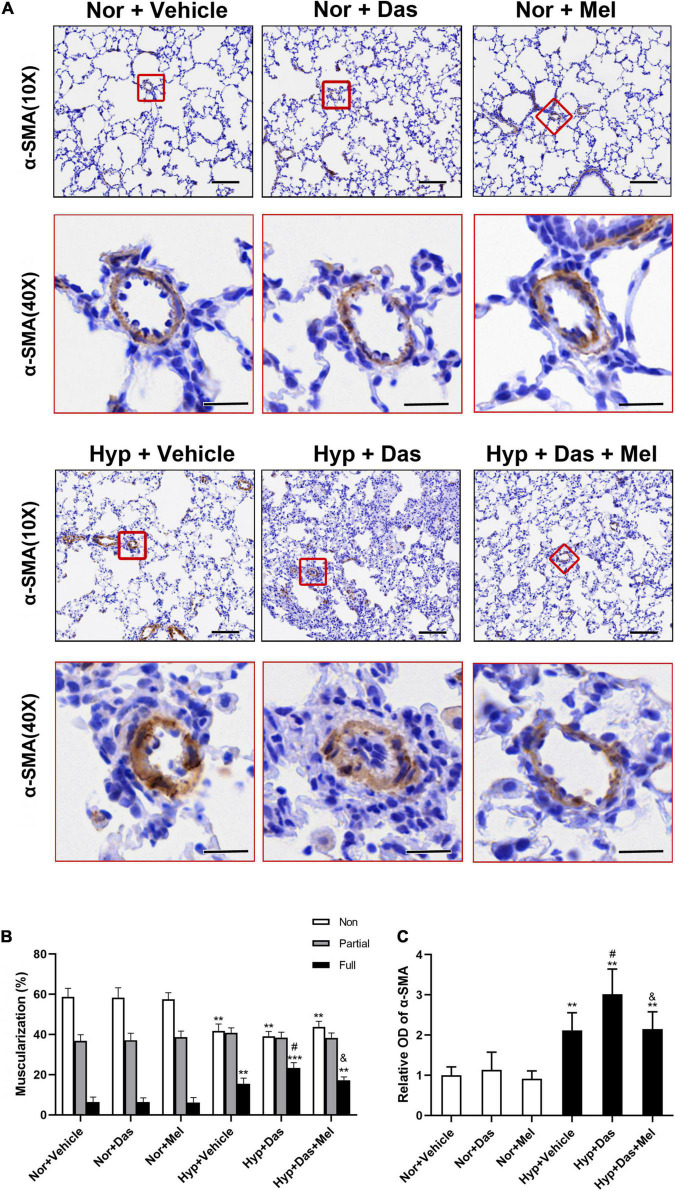
Melatonin attenuates pulmonary arteries muscularization induced by dasatinib under hypoxia. α-SMA (smooth muscle actin) immunohistochemical staining of pulmonary arterioles (original magnification ×10, scale bars: 100 μm, and original magnification ×40, scale bars: 20 μm) **(A)**. The quantitative analysis of the proportion of muscularized pulmonary arteries (sized 25–100 μm) **(B)**. The quantitative analysis of OD value of α-SMA immunoreactivity in pulmonary arterioles **(C)**. Values are means ± SEM, ***P < 0.001, **P < 0.01 compared with Nor + Vehicle group. *^#^P* < 0.05 compared with Hyp + Vehicle group. *^&^P* < 0.05 compared with Hyp + Das group (*n* = 6).

Similarly to the observed histological changes, non-and full muscularization vessels showed significant differences between normoxic and hypoxic groups. Also, the percentage of lung full muscularization arterioles in Hyp + Das group rats was higher than that in Hyp + Vehicle group rats. However, the percentage of lung full muscularization arterioles in Hyp + Das + Mel group rats was lower than that in Hyp + Das group rats (Figures 3A,B). α-SMA positive-area of pulmonary arterioles was significantly increased in Hyp + Das group rats, whereas this increment of the α-SMA positive area was lower in Hyp + Das + Mel group rats (Figures 3A,C).

**FIGURE 4:**
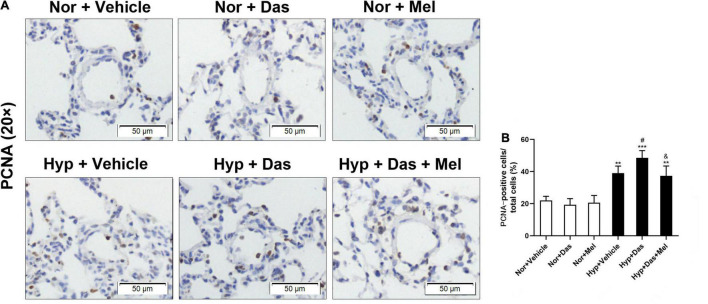
PCNA staining of pulmonary arterioles (original magnification × 20, scale bars: 50 μm) **(A)**, bar graph shows that the number of proliferating cell nuclear antigen (PCNA)-positive cells relative to the total smooth muscle cells in the medial wall of pulmonary arterioles (%) **(B)**. Values are means ± SEM, ***P < 0.001, **P < 0.01 compared with Nor + Vehicle group. *^#^P* < 0.05 compared with Hyp + Vehicle group. *^&^P* < 0.05 compared with Hyp + Das group (*n* = 6).

Additionally, we analyzed the cell proliferation in the medial wall of small pulmonary vessels. As Figure 4 showed that the percentage of PCNA-positive cells in Hyp + Das group was higher than that in Hyp + Vehicle group, the increased cell proliferation was diminished in Hyp + Das + Mel group rats (Figures 4A,B).

### Melatonin Ameliorated Dasatinib-Aggravated Pulmonary Oxidative Damage Under Hypoxia in Rats

**FIGURE 5:**
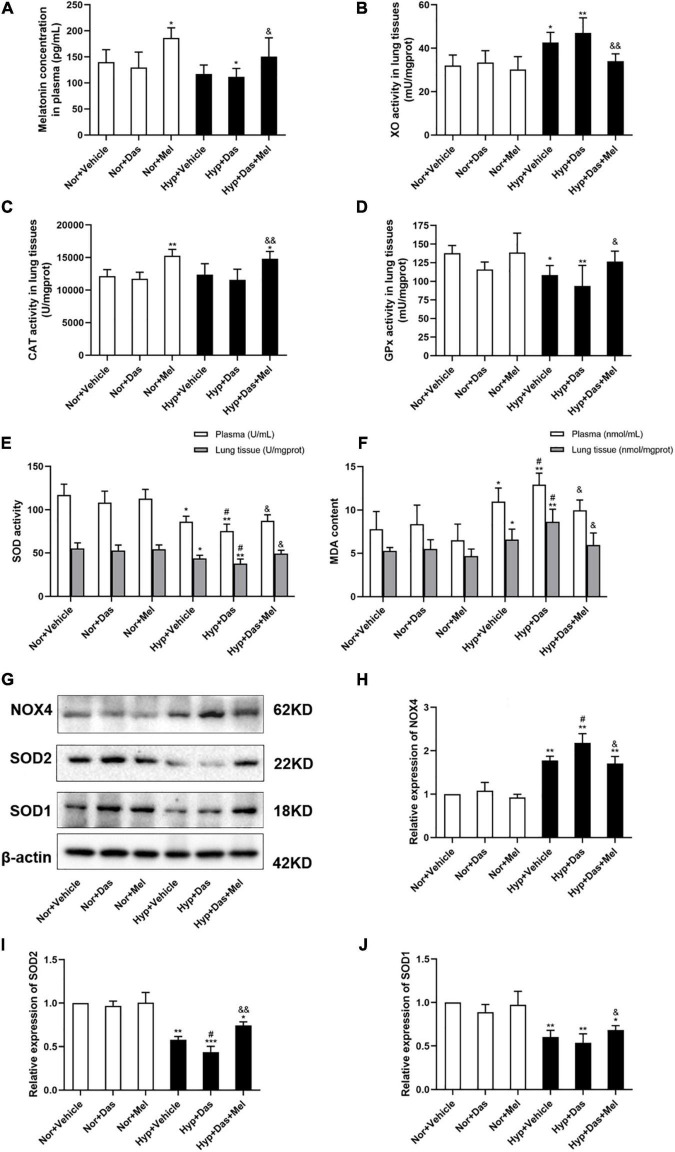
Melatonin attenuates pulmonary oxidative stress induced by dasatinib under hypoxia. The melatonin concentration in rat plasma **(A)**, xanthine oxidase (XO) activity in rat lung tissues **(B)**, Catalase (CAT) activity in rat lung tissues **(C)**, Glutathione peroxidase (GPx) activity in rat lung tissues **(D)** (n = 6). The activity of superoxide dismutase (SOD) in rat plasma and lung tissues **(E)**, Malondialdehyde (MDA) content in plasma and rat lung tissues **(F)** (*n* = 6). Protein expression of NOX4, SOD2, and SOD1 in rat lung tissues **(G)**. Bar graph shows the statistic results of NOX4 **(H)**, SOD2 **(I)**, and SOD1 **(J)** protein levels (*n* = 3). Values are means ± SEM, ***P < 0.001, **P < 0.01, *P < 0.05 compared with Nor + Vehicle group. *^#^P* < 0.05 compared with Hyp + Vehicle group. *^&&^P* < 0.01, *^&^P* < 0.05 compared with Hyp + Das group.

The melatonin concentration in plasma of Hyp + Das group was lower compared to Nor + Vehicle group, however the melatonin concentration in plasma of Hyp + Das + Mel group was markedly increased, relative to Hyp + Das group (Figure 5A).

Pro-oxidant enzyme XO plays a critical role in the regulation of the oxidant levels in the vasculature of PH. To determine the anti-oxidative effect of melatonin, XO activity in lung tissues was analyzed. Results showed that pulmonary XO activity of Hyp + Das group and Hyp + Vehicle group was higher compared to Nor + Vehicle, melatonin treatment reduced the levels of XO in lung tissues (Figure 5B).

The dysregulation of antioxidant enzymes implicated in the etiology of PH, therefore, the activity of CAT, GPx, and SOD were detected. The CAT activity in lung tissues of Nor + Mel group and Hyp + Das + Mel group was higher compared to Nor + Vehicle group (Figure 5C). Pulmonary GPx activity in Hyp + Vehicle group and Hyp + Das group was lower compared to Nor + Vehicle, melatonin treatment reversed the levels of GPx in lung tissues (Figure 5D). Plasma and pulmonary SOD activity in Hyp + Das group rats were lower compared to Hyp + Vehicle (Figure 5E). However, these above changes were reversed in Hyp + Das + Mel group rats (Figures 5C–E).

MDA is formed as an end product of lipid peroxidation and acts as a marker of endogenous lipid peroxidation. Our results showed that plasma and pulmonary MDA content in Hyp + Das group rats were higher compared to Hyp + Vehicle group, these changes were inhibited in Hyp + Das + Mel group rats (Figure 5F).

Furthermore, as Figures 5G–J shown that pulmonary SOD2 expression in Hyp + Das group rats was lower compared to Hyp + Vehicle group, NOX4 expression was higher than Hyp + Vehicle group, but SOD1 expression showed no significant change between Hyp + Das group and Hyp + Vehicle group. The above changes were reversed by melatonin treatment.

### Melatonin Protected Human Pulmonary Endothelial Cells (HPMECs) From Dasatinib-Induced Apoptosis via Reducing ROS Production Under Hypoxia

**FIGURE 6:**
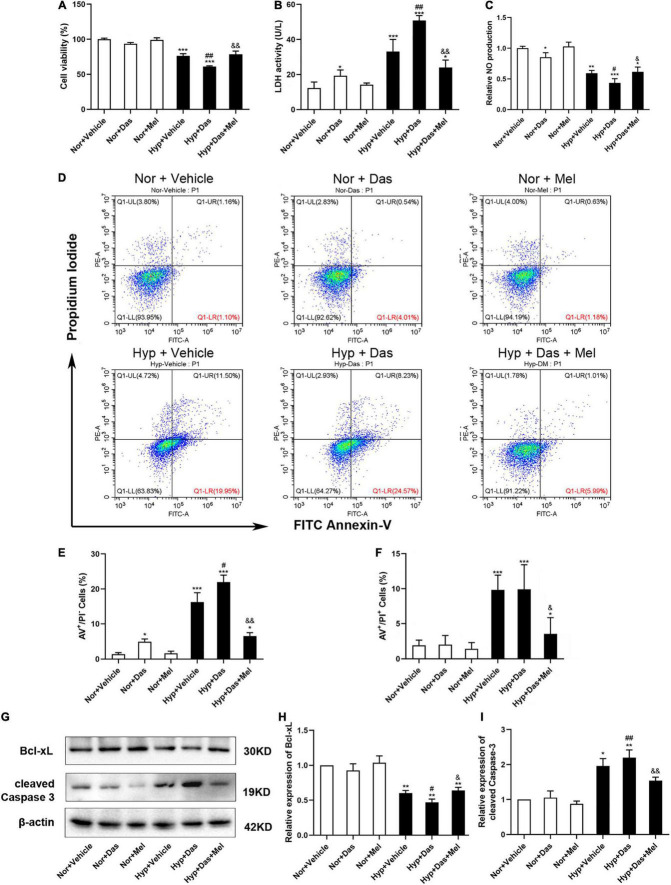
Melatonin protects human pulmonary endothelial cells (HPMECs) from dasatinib-induced apoptosis under hypoxia. Cell viability detected with cell counting kit-8 assay **(A)**, LDH activity in the medium **(B)** (*n* = 6), Relative NO production **(C)** (*n* = 4), Representative FACS dot plots of annexin V (AV) and propidium iodide (PI) dual labeling in HPMECs **(C)**, quantification of AV and PI dual labeling in HPMECs **(D–F)**. Protein expression of Bcl-xL and cleaved Caspase-3 in HPMECs **(G)**. Bar graph shows the statistic results of Bcl-xL, cleaved Caspase-3 protein levels **(H,I)** (*n* = 3). Values are means ± SEM, ***P < 0.001, **P < 0.01, *P < 0.05 compared with Nor + Vehicle group. *^##^P* < 0.01, *^#^P* < 0.05 compared with Hyp + Vehicle group. *^&&^P* < 0.01, *^&^P* < 0.05 compared with Hyp + Das group.

We next assessed that melatonin attenuated pulmonary EC dysfunction which induced by dasatinib under hypoxia via studying its direct effect on HPMECs *in vitro*. The cell viability of HPMECs in Hyp + Das group was diminished than Hyp + Vehicle group, 10 μM melatonin significantly enhanced HPMEC viability effectively under dasatinib treatment and hypoxia (Figure 6A). LDH activity of HPMEC medium was higher in Hyp + Das group than Hyp + Vehicle group (Figure 6B). NO generation of HPMECs in Hyp + Das group was decreased compared to Hyp + Vehicle group, whereas melatonin attenuated the change (Figure 6C). AV-positive/PI-negative cells were elevated in the Hyp + Das group, whereas melatonin attenuated dasatinib- and hypoxia- induced apoptosis of HPMECs and protected endothelial cells from apoptosis in response to injury (Figures 6D–F). Cleaved caspase-3 expression was higher, and Bcl-xL expression was lower in Hyp + Das group than Hyp + Vehicle group, melatonin treatment conspicuously reversed the above-mentioned changes of HPMECs (Figures 6G–I).

**FIGURE 7:**
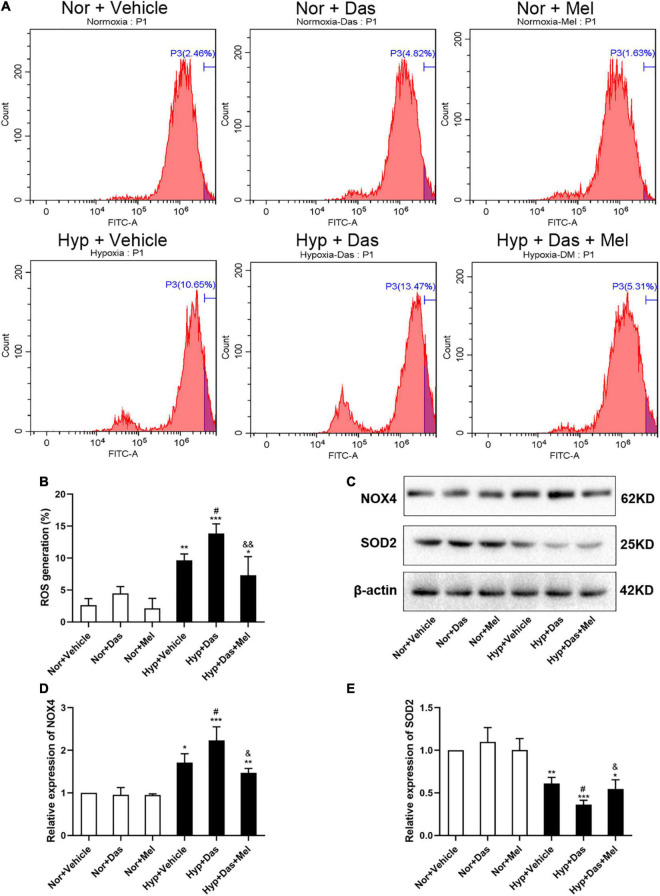
Melatonin protects HPMECs from dasatinib-induced oxidative injury under hypoxia. Level of intracellular ROS generation **(A)**. A summary of flow cytometry analyses of HPMECs stained with 2,7-dichlorodihydrofluorescein-diacetate (DCFH-DA) **(B)**. Protein expression of NOX4 and SOD2 in HPMECs **(C)**. Bar graph shows the statistic results of NOX4 and SOD2 protein levels **(D,E)**. Values are means ± SEM, ***P < 0.001, **P < 0.01, *P < 0.05 compared with Nor + Vehicle group. *^#^P* < 0.05 compared with Hyp + Vehicle group. *^&&^P* < 0.01, *^&^P* < 0.05 compared with Hyp + Das group (*n* = 3).

To further explore the possible underlying mechanism, we examined oxidative stress level in HPMECs. ROS generation of HPMECs was higher in Hyp + Das group than Hyp + Vehicle group (Figures 7A,B). NOX4 protein expression was higher in Hyp + Das group than Hyp + Vehicle group (Figures 7C,D). Protein expression of SOD2 was lower in Hyp + Das group than Hyp + Vehicle group (Figures 7C,E). Melatonin treatment significantly inhibited the above-mentioned changes of HPMECs (Figure 7).

### Melatonin Inhibited Rat PASMCs Proliferation Induced by Dasatinib Under Hypoxia via Reducing ROS Production

**FIGURE 8:**
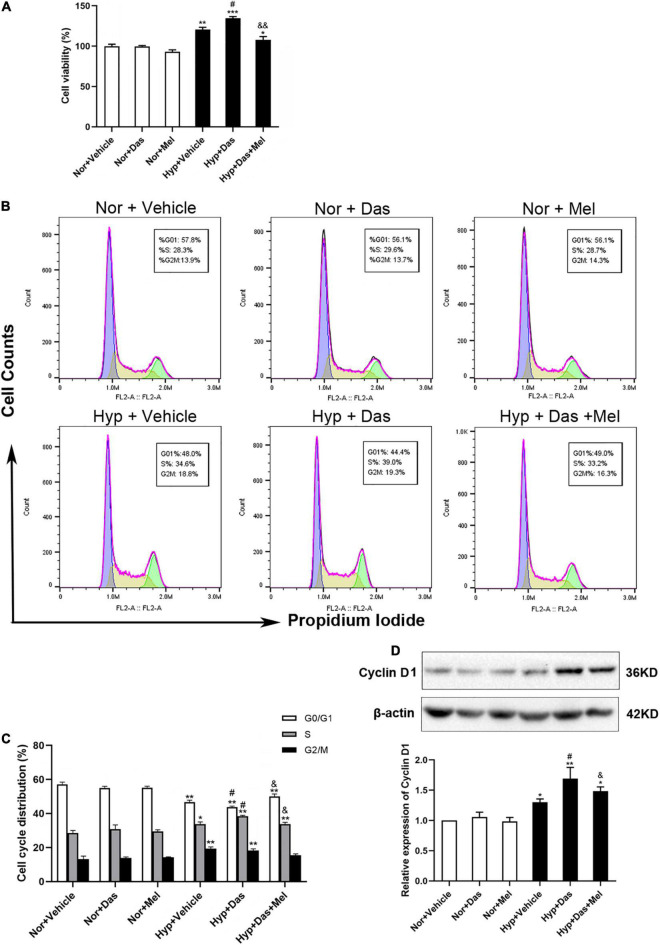
Melatonin arrests cell cycle process acceleration of pulmonary artery smooth muscle cells (PASMCs) induced by dasatinib under hypoxia. Viability of PASMCs pretreatment with melatonin or vehicle induced by dasatinib under hypoxia for 24 h was determined by cell counting kit-8 (CCK8) assay **(A)**. Representative FACS dot plots of cell cycle in PASMCs **(B)**, the percentage of cells at each phase of cell cycle was quantified **(C)**. Protein expression of Cyclin D1 in PASMCs **(D)**. Values are means ± SEM, ***P < 0.001, **P < 0.01, *P < 0.05 compared with Nor + Vehicle group. *^#^P* < 0.05 compared with Hyp + Vehicle group. *^&&^P* < 0.01, *^&^P* < 0.05 compared with Hyp + Das group (*n* = 3).

Excessive PASMCs proliferation is considered a hallmark of pulmonary vascular remodeling in PH. Hence we detected the role of dasatinib on PASMCs *in vitro*. First, we isolated the PASMCs from rats. Then we investigated the cell viability of PASMCs induced by dasatinib under normoxia or hypoxia. CCK-8 assay showed dasatinib under hypoxia significantly increased the cell viability compared to hypoxia alone, 10 μM melatonin treatment effectively inhibited the proliferation of PASMCs induced by dasatinib under hypoxia (Figure 8A).

Next, we determined whether the inhibition of dasatinib-aggravated PASMCs proliferation by melatonin was correlated with cell cycle arrest. Dasatinib- and hypoxia-treatment increased the percentage of PASMCs in the S phase with a concomitant decrease in the G0/G1 phase and G2/M phase, whereas melatonin reversed the above-mentioned changes (Figures 8B,C). Additionally, we detected the expression of cyclin D1, the pivotal protein of cell cycle in PASMCs. Cyclin D1 expression was higher in Hyp + Das group than Hyp + Vehicle group PASMCs, however, melatonin remarkably decreased the expression of cyclin D1, which was induced by dasatinib under hypoxia (Figure 8D).

**FIGURE 9:**
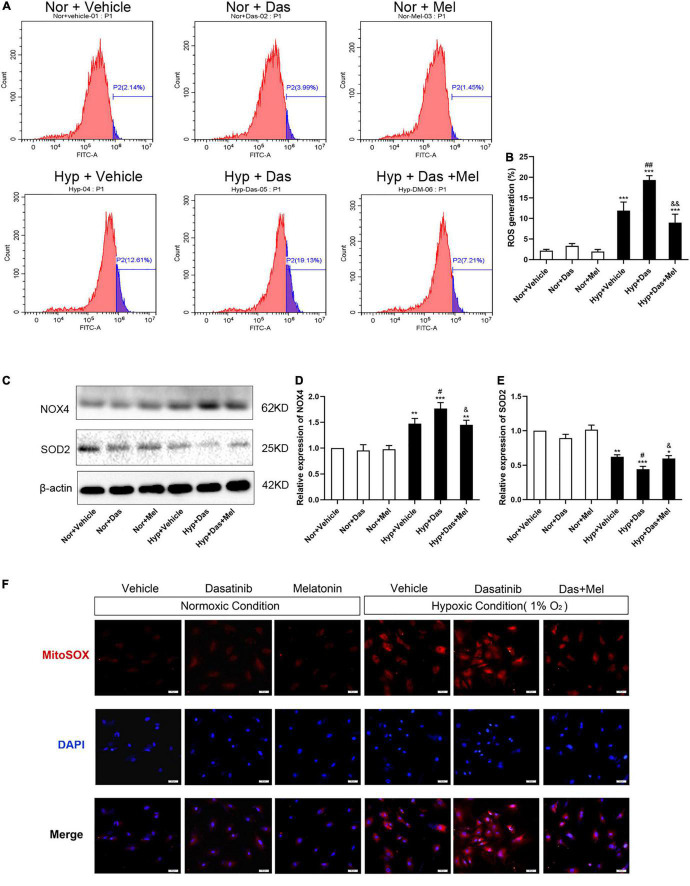
Melatonin attenuates oxidative stress of pulmonary artery smooth muscle cells (PASMCs) induced by dasatinib under hypoxia *in vitro*. Flow cytometry analyses of PASMCs stained with 2, 7-dichlorodihydrofluorescein diacetate (DCFH-DA) **(A)**. A summary of intracellular ROS generation **(B)** (*n* = 3). Protein expression of NOX4, SOD2 in PASMCs **(C)**. Bar graph shows the statistic results of NOX4, SOD2 protein levels **(D,E)**. Mitochondrial ROS with MitoSOX (in red), DAPI was used to label the nucleus (in blue), and the merged images of MitoSOX and DAPI were shown on the right (original magnification ×20, scale bars: 50 μm) **(F)**. Values are means ± SEM, ***P < 0.001, **P < 0.01, *P < 0.05 compared with Nor + Vehicle group. *^##^P* < 0.01, *^#^P* < 0.05 compared with Hyp + Vehicle group. *^&&^P* < 0.01, *^&^P* < 0.05 compared with Hyp + Das group (*n* = 3).

To gain more insight into cellular and molecular mechanisms underlying dasatinib-aggravated PASMCs proliferation, the impact of melatonin on oxidative stress of primary rat PASMCs was assessed. ROS generation and NOX4 expression were higher in Hyp + Das group than Hyp + Vehicle group PASMCs (Figures 9A–D). Protein expression of SOD2 was lower in Hyp + Das group PASMCs than Hyp + Vehicle group PASMCs (Figures 9C,E). Melatonin treatment significantly inhibited above-mentioned changes of PASMCs (Figures 9A–E). Except for NADPH oxidase, mitochondrial ROS is another crucial element that leads to oxidative stress (31–33). Similarly to the protein expression of SOD2 change, dasatinib *in vitro* treatment under hypoxia led to an increase in mitochondrial ROS production, melatonin reversed the increase in dasatinib-aggravated mitochondrial ROS production of PASMCs under hypoxia (Figure 9F).

### Melatonin Reversed HIF1-α Overexpression and Repressed HIF1-α Protein Stability Induced by Dasatinib Under Hypoxia

**FIGURE 10:**
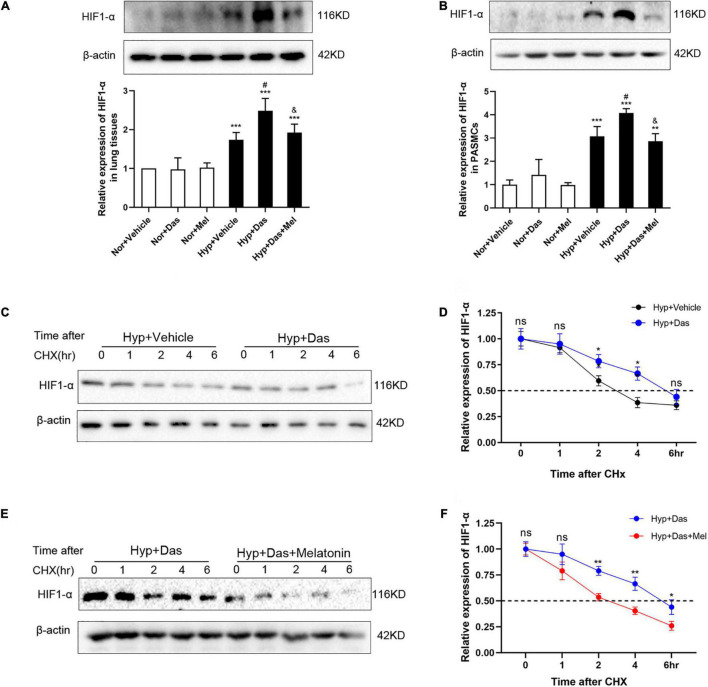
Melatonin repressed HIF1-α protein expression induced by dasatinib under hypoxia. Protein expression of HIF1-α in rat lung tissues **(A)**. Protein expression of HIF1-α in PASMCs **(B)**. Values are means ± SEM, ***P < 0.001, **P < 0.01 compared with Nor + Vehicle group. *^#^P* < 0.05 compared with Hyp + Vehicle group. *^&^P* < 0.05 compared with Hyp + Das group. PASMCs are exposed to dasatinib or vehicle **(C,D)**, dasatinib or dasatinib with melatonin **(E,F)** under hypoxia treatment for 24 h before the addition of cycloheximide (12.5 μg/mL). Lysates were harvested at the indicated time points, HIF-1α expression was monitored by Western blotting, and densitometry was used to determine the rate of decay. Values are means ± SEM, **P < 0.01, *P < 0.05, ns indicates non-significant (*n* = 3).

HIF1-α (hypoxia-inducible factor 1-α) plays an essential role in the development of pulmonary vascular remodeling during PH (9, 34, 35). Results showed that HIF1-α was higher in Hyp + Das group than Hyp + Vehicle group, and melatonin reversed the dasatinib- and hypoxia-induced up-regulation of HIF1-α both in lungs of rats and PASMCs (Figures 10A,B). Next, we examined whether melatonin influenced the protein stability of HIF1-α with the treatment of dasatinib under hypoxia. As shown in Figures 10C–F, by using cycloheximide (CHx), we found that melatonin repressed HIF1-α protein stability in PASMCs with dasatinib treatment under hypoxia.

## Discussion

In the present study, the dasatinib-aggravated hypoxic PH model exhibited significant structural remodeling of small pulmonary vessels and elevated RVSP as in the previous report (8), suggested that dasatinib caused the development of PH may through suffering the “second hit” (such as environmental and/or genetic factors). Additionally, dasatinib could induce ECs apoptosis, also cause SMCs proliferation *in vitro*. Notably, melatonin supplementation inhibited dasatinib-aggravated elevation of RVSP, Fulton index, cardio output, WT% and WA% under hypoxia *in vivo*, prevented pulmonary ECs damage, and SMCs proliferation from the induction of dasatinib, suggesting that melatonin attenuated dasatinib-aggravated hypoxic PH in rats.

Obtaining a more comprehensive understanding of the redox pathways and the mechanisms of pro-oxidants/anti-oxidants is significant to develop drugs to prevent vascular remodeling in PH (14–17). It is important to explore the imbalance of anti-oxidases/pro-oxidases in dasatinib-associated PH. CAT, GPx, and SOD are generally considered to be the dominant hydrogen peroxide-scavenging enzymes in the lung. GPx can also act on peroxides other than H_2_O_2_, such as promoting the reduction of fatty acid hydroperoxides (17, 36, 37). Particularly SOD is the only enzymatic system decomposing superoxide radicals to hydrogen peroxide. SOD serves to catalyze the rapid conversion of superoxide radical to hydrogen peroxide, balances oxygen radicals, and removes stress from oxidation state (38). It has been known that SOD2 protein expression decreases in lung tissues of severe PH patients (39). SOD2 deficiency initiates PH by impairing redox signaling, activating HIF1-α and creating proliferative PASMCs (40). In the present study, dasatinib inhibited SOD and GPx activity, SOD2 expression in rat lungs under hypoxia, while melatonin reversed the above changes induced by dasatinib.

Furthermore, XO is the enzyme that catalyzes the conversion of hypoxanthine to xanthine and xanthine to uric acid with concomitant generation of anion superoxide. Previous studies have shown that the treatment of melatonin did not affect the protein expression of XO in PH newborn sheep, but decreased the activity of XO (41). One mechanism by which oxidants may cause pulmonary injury is lipid peroxidation. MDA level is elevated in serum and plasma of PH patients (42, 43). NOX4, an essential source of intracellular ROS production, is thought to play a vital role in developing elevated pulmonary artery resistance and pressure (28, 29, 44–47). Our results showed that melatonin attenuated XO activity, MDA content, NOX4 protein expression induced by dasatinib under hypoxia. Together, these observations suggested that melatonin abrogated dasatinib-aggravated pulmonary vascular remodeling through inhibiting pulmonary oxidative stress in rats.

Endothelial dysfunction plays an important role in the initiation and progression of pulmonary vascular remodeling in PH, irrespective of disease origin (48, 49). Pulmonary artery ECs in an apoptotic state liberate a variety of cytokines and ROS, excessively consume NO, which subsequently stimulate endothelial dysfunction (8, 50). Increased level of ROS and apoptosis has been proved to be closely associated to oxidative stress, which is a key factor contributing to pulmonary vascular remodeling. In the present study, melatonin protected HPMECs from dasatinib-induced apoptosis and reduced oxidative stress level via elevating SOD2 expression, diminishing NOX4 expression, and inhibiting ROS generation under hypoxia. The above results suggested that melatonin alleviated dasatinib-aggravated pulmonary vascular remodeling via up-regulating anti-oxidant enzyme, reducing pro-oxidant level, improving NO production and ROS clearance, ameliorating oxidative damage, and inhibiting apoptosis in ECs.

Abnormal proliferation of SMCs is considered a major mechanism underlying the development of pulmonary vascular remodeling in PH. Even if the current medications of PH predominantly targeting vasoconstriction can provide symptomatic relief, they are unable to attenuate excessive PASMCs proliferation and occlusive vascular remodeling (51–54). In the present study, we found that melatonin significantly attenuated the enhanced α-SMA immunoreactivity, the increased full muscularization arterioles, and the elevated PCNA-positive cells of pulmonary arteries induced by dasatinib under hypoxia *in vivo*, suggesting that melatonin remarkably blunted the progressive media thickening induced by dasatinib under hypoxia. It has been reported that dasatinib induces vascular remodeling, involving the progressive thickening of small pulmonary arteries (8). Nevertheless, there is no study about the effect of dasatinib on proliferation of SMCs in PH. Herein, it was found that dasatinib could directly induce PASMCs proliferation via increasing cell viability and arresting cell cycle process. As one of the key regulators for G1/S transition in the cell cycle, cyclin D1 is up-regulated in excessively proliferative PASMCs by hypoxia, resulting in pulmonary vascular remodeling and PH (55, 56). Notably, our results indicated that melatonin reversed the up-regulation of cyclin D1 and led to cell cycle G0/G1 phase arrest induced by dasatinib under hypoxia in primary rat PASMCs. Furthermore, melatonin mitigated ROS and mitoSOX generation, in accord with NOX4 down-regulation and SOD2 up-regulation under dasatinib- and hypoxia- induction. It has been confirmed that ROS promotes SMCs proliferation both directly and indirectly by inducing auto/paracrine growth mechanisms (57, 58). Taken together, the current results suggested that melatonin mitigated dasatinib-aggravated PASMCs proliferation via promoting the anti-oxidant capacity and scavenging free radicals.

HIF1-α protein expression can be stabilized by ROS(9, 16), the stability of HIF1-α is considered an intrinsic pathogenic determinant in PH (9, 59). SMC-specific *HIF1-*α knockout mice exert less pulmonary vascular remodeling and PH after chronic hypoxia induction (11). Recent studies have implicated that aberrant activation of HIF1-α contributes to PASMCs proliferation, pulmonary vascular remodeling and PH pathogenesis (30, 60). The present study demonstrated that melatonin repressed dasatinib-induced HIF1-α protein stability in primary rat PASMCs under hypoxia. Further studies may focus on the exact mechanism of melatonin on protein stability of HIF1-α in dasatinib-induced PASMCs. The effect of melatonin on ubiquitination and degradation of HIF1-α, regulating HRE sites and revealing associated target genes may be involved in, which were induced by dasatinib is required to be researched.

One of the limitations of our study is that we do not actually know if the effects of melatonin are receptor-mediated. MT1 and MT2 receptors are present in pulmonary vessels of animals (61, 62). Previous study has shown that the MT1 receptor is suggested to be playing a role in the gene regulatory action by melatonin, the MT2 receptor is reported to be involved in mediating vasorelaxation (63). Hence, whether the effects of melatonin are via receptor dependent or independent mechanism is needed to be explored.

**FIGURE 11:**
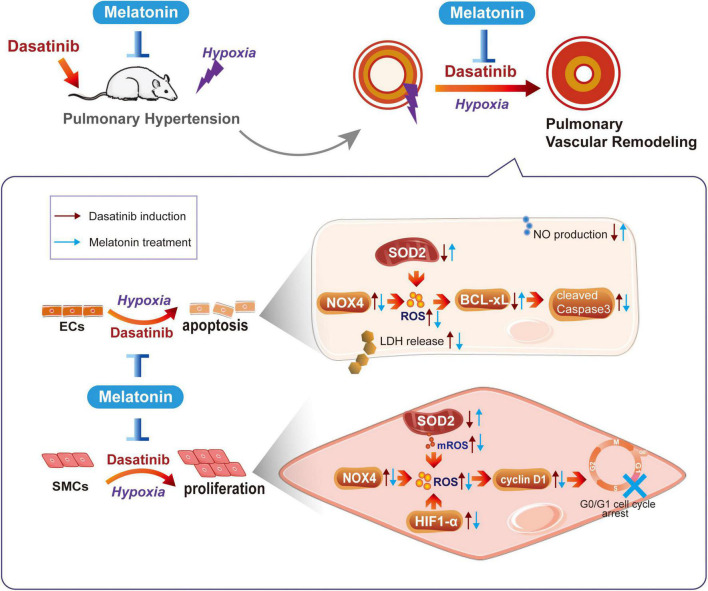
Schematic diagram of this study. Melatonin attenuates dasatinib-aggravated hypoxic pulmonary hypertension via inhibiting pulmonary vascular remodeling.

In conclusion, melatonin significantly attenuates dasatinib-induced PH by inhibiting pulmonary vascular remodeling in rats. The mechanisms probably involved protecting ECs through inhibiting oxidative damage and apoptosis, and inhibiting abnormal proliferation of SMCs through decreasing HIF1-α protein stability, oxidative stress, and arresting cell cycle (Figure 11). Our findings may suggest that melatonin has potential clinical value as a therapeutic approach to alleviate dasatinib-aggravated hypoxic PH.

## Data Availability Statement

The original contributions presented in the study are included in the article, further inquiries can be directed to the corresponding authors.

## Ethics Statement

The animal study was reviewed and approved by Animal Experimental Ethics Committee of Dalian Medical University.

## Author Contributions

RW, JH, JY, HL, and YY conceived and designed the experiments. RW, JH, MG, LL, YZ, YL, DW, QT, NW, and LW collected, analyzed, and interpreted the experimental data. RW and JP drafted the manuscript. All authors revised the manuscript critically and approved the final version.

## Conflict of Interest

The authors declare that the research was conducted in the absence of any commercial or financial relationships that could be construed as a potential conflict of interest.

## Publisher’s Note

All claims expressed in this article are solely those of the authors and do not necessarily represent those of their affiliated organizations, or those of the publisher, the editors and the reviewers. Any product that may be evaluated in this article, or claim that may be made by its manufacturer, is not guaranteed or endorsed by the publisher.
